# The Intervention of the State in Medical Affairs

**Published:** 1883-12

**Authors:** 


### THE INTERVENTION OF THE STATE IN MEDICAL AFFAIRS.

The questions which have lately been suggested touching the relations of medicine and medical men to the State, are daily acquiring more interest. They have given rise to a number of issues which at the outset could scarcely have been considered as probable results of the first laws framed with a view to the regulation of the practice of medicine. Some of these issues we have undertaken to discuss in previous numbers of this journal. The whole subject will acquire an added interest for those who take the pains to read Professor Huxley's address, lately delivered at the annual distribution of prizes at the London Hospital Medical College, and printed in the *British Medical Journal* of October 18, 1883. The name of the distinguished speaker, no less than the interest attaching to his subject—"The Intervention of the State in the Affairs of the Medical Profession"—ensures for both a respectful consideration in all English-speaking countries.

Prof. Huxley practically presents three questions: 1. Why should the State interfere with medicine and the medical profession? 2. If there be ground for such intervention, what should be its extent? 3. If such intervention be right and proper, how can it best be carried into effect? The answers which he proposes to these several inquiries, seem to us eminently wise and judicious; and some of the commentaries they suggest are interesting to us on this side of the Atlantic.

With respect to the first question, the speaker took the ground that it was not the duty of the State to take medical charge of the public, to protect it against incompetent persons in general, and in particular against quacks and impostors. He thought that it was more wholesome for the public to take care of itself, wherever it can do so, in this as well as all other matters; and that, on the whole, there should be no interference with the liberty of each person to do that which he likes, when he does not interfere with others. The actual impossibility (which all reasonable men must admit, and of which we, in the State of Illinois, have seen the most conspicuous and notorious examples) of absolutely prohibiting the practice of medicine by people not specially qualified for it, is freely admitted by Professor Huxley. The old lady who orders a cranberry poultice, the druggist who undertakes to relieve the pain of an aching tooth or of any other afflicted organ of the body, will enjoy these peculiar pastimes in the face of all legal restrictions. The charlatan will do no less, and always more. *Facilis descensus Averni.* He has smiled equally at the British, Gallic and Teutonic barriers erected to bar his progress. Where there is a throne, there will always be a pretender. The shadow will always follow the substance. We shall always have him with us. Whether the truth or its counterfeit shall prevail, is determined not by a national legislature, but by the extent of the diffusion of knowledge among the people whose feeblest index that legislature often is.

Professor Huxley, however, strikes the key-note of the whole question when he points to the death of the individual citizen as the important moment which the State should officially recognize. Here the volition of the individual ceases. In civil and criminal causes, the law should be able to have recourse to persons qualified to give expert evidence. Here lies the justification of the intervention of the State. It says to one class of men, "Practice medicine, if you like, on any basis, on any theory, whether you are qualified or not qualified;" and, to another class, "Consult whom you choose, pay whom you will for attendance upon you in your illnesses; but before I can receive from you a certificate of death, before I can appoint any of you to my civil or military service, the standard of my qualifications must be attained. The giver of the certificate, the incumbent of the office, must be those whom I can recognize as fit for such service." In brief, the State intervenes only at those points where the

State touches the individual. With the old woman's elder-flower tea, with the druggist's cough panacea, with the quack's startling announcement of the discovery of the cure of consumption, the State has no more to do than with the numerous patent religious enterprises of the day in which we live.

Justifying, on this admission of its necessity, the intervention of the State to this extent in medical matters, the speaker went on to briefly describe the ancient sources of the license to practice in his country, including the universities, guilds, corporations, and even the Archbishop of Canterbury, who has lately retired from the business. The year 1858, the date of the Medical Act, barely twenty-five years ago, was the relatively recent beginning of the present improved condition of affairs. We, of this country, looking upon the praiseworthy condition of the standards of medical education in Great Britain cannot certainly ascribe all their excellence to the changes effected in the licensing bodies by the Act of 1858. We cannot but be sure that the average British practitioner of to-day is of the quality that he is, not because of the improvements since 1858, but in the face of the "fine old crusted abuses" and of the caricatures of a respectable source of power and authority, which existed before that year.

Certain admissions here made by the distinguished speaker are very significant. He says, speaking of the condition of affairs, not then but now: "Unfortunately, it is equally certain that there remain two or three black sheep — licensing bodies which simply trade upon their privilege, and sell the cheapest wares that they, for shame's sake, can supply to the bidder." Here is a remarkable admission from the lips of one who knows of what he speaks, and who speaks with authority. No news indeed for Americans well informed as to medical matters in Great Britain! It is an admission which should afford us neither satisfaction nor encouragement. Still, as a matter of fact, it teaches us in a most unmistakable manner, that in that country where the State does visibly and powerfully interfere in medical affairs, the same great field is left vacant where, as in this country, avarice on the one hand and ignorance on the other, can set up their unscrupulous mart and indulge in their disgraceful traffic!

Nor is this all. Professor Huxley goes on to show that there is another defect in the existing system, as a result of which the licensing power may authorize practice on the basis of an acquaintance with either medicine or surgery alone. The surgeon, for example, totally unacquainted with either medicine or midwifery, may be thus accredited to the public, and successfully embark upon the deep of pecuniary profit with a miserably inadequate professional outfit.

The report of the commission having in view the correction of these obvious defects, is characterized by Professor Huxley as an "elaborate, complex, and rather clumsy contrivance, of superseding a number of institutions which up to this time have done their work exceedingly well and are doing it better every day." He proceeds to deprecate any interference with vested interests, or with the prestige of institutions which have been and still are extremely valuable. The admirable way in which these have done their work, has wrought an effect which has been on several occasions described by ourselves, as the natural result of the forces which operate in this field when they are untrammeled by law. The speaker proceeded to show that henceforward, as heretofore, students would throng to obtain their qualification of those corporate bodies which had acquired a great and noble prestige ; while those who had neglected their duties and "in one or two cases had actually disgraced themselves, would sink into oblivion and come to a happy euthanasia, in which their misdeeds and themselves would be entirely forgotten." We should seek in vain to describe more aptly the condition of affairs in this State, where the institutions of medical learning, which were in existence and had acquired an honorable prestige before the establishment and intervention of a State law touching their affairs, are to-day still existing, still adding to their prestige, still honorably discharging their high functions, not because of the law, but in the face of a law which, in certain particulars at least, has worked toward the limitation of their power.

Referring to the question of preliminary education and preliminary examination of students, previous to the four years' curriculum in medicine, we are scarcely surprised to hear the lecturer say, that the young Briton presents himself, in all probability absolutely ignorant of the existence of science, having never heard of chemistry or of physics, and without the smallest conception of the outlines of pathological research. The remedy proposed for this condition of affairs, is not what many of the ardent advocates of reform in the standards of American medical education would either suggest or advocate. It is not to make the examination more rigorous, to exclude a larger number of applicants, to reject a larger number of those who are determined in their minds, either by the nobler and better or by the cheaper and meaner avenues, to enter the medical profession. Professor Huxley is indeed sorry to believe that the general education examination is "entirely futile." His plan is, admitting it to be practically hopeless to extend the period of professional study beyond the twenty-second year, to prolong the course, as it were, backward. After the sixteenth or the seventeenth year of life, the time might be usefully employed, not in an education preliminary to the study of medicine, but as a matter of fact in that study itself. Thus, elementary physics, chemistry, biology, anatomy, physiology, physiological chemistry, and physiological physics might be then profitably studied, going over a field which has become so large that it is utterly impossible that any man in it should be, under the present system, competently taught by a competent teacher.

Professor Huxley's suggestion that these so-called "institutes of medicine" should be taught in the earlier period of study in one or more separate institutions; and the more practical branches later, in the medical schools of his own country, is not only practically valuable itself, but full of suggestiveness to those who are connected with American schools. The latter are multiplying yearly under the stimulus of the ambition of men anxious to become preceptors in medicine. Many of the latter reside in towns of relatively small population and inferior medical opportunities. Among existing institutions, it is needless to add that there are those of a respectable character and legitimate authority, whose situation and opportunities are greatly inferior to the standard of requirements for a medical university of high-class position. The benefits that would assuredly result from a division of labor between these several institutions, it would be difficult to exaggerate. Let those whose facilities are inferior and whose resources are inadequate, devote themselves exclusively to the preliminary instruction of students in these so-called "institutes of medicine;" and the larger schools, situated in the great metropolitan centers, with unrivaled facilities and practically unlimited clinical material, will willingly give themselves especially to the practical teaching connected with disease and injury, which is the great and pressing requirement of him who would be a practitioner of medicine. The medical degree would naturally be conferred by the latter. The realization of such a scheme, which should not be hastily pronounced either visionary or impracticable, would inure to the benefit of both classes of schools, as also of the general profession. It would prolong the period of medical study, without insisting that the faithful, painstaking, and ambitious American student should pass the best portion of his earlier manhood within the shadow of a university, without repelling from the ranks of the profession some of those well qualified by nature to enter them, and without inflicting a damage upon corporate bodies which are already, with credit to themselves and with usefulness to the world at large, improving by all the means which wealth and knowledge can command, the character and requirements of the medical college curriculum in America.

---

In the last issue of this JOURNAL we copied an item from the *Peoria Medical Monthly*, in which reference was made to a handbill, ostensibly issued by a person by the name of J. B. Findley, announcing that Professor J. E. Harper, of the Chicago College of Physicians and Surgeons, was about to visit Bloomfield, Iowa, and soliciting the attendance there of patients affected with disorders of the eye and ear.

We take special pleasure in saying that after a careful investigation of all the facts in this case, it has become perfectly clear, that Professor Harper is and was entirely innocent of any attempt whatever to advertise himself in this manner; that he had no collusion or sympathy with Findley in the printing and circulation of this handbill; and that he has our sympathy since he has really suffered from the well meant but exceedingly ill directed intentions of a friend.

We are pleased to add that Professor Harper has the full esteem and confidence of the profession in this city, and can be trusted to engage in no attempts unworthy of his high reputation as a physician, observing with strict regard all the proprieties of the profession.

---

### HYPODERMIC INJECTIONS OF ETHER IN CASES OF PNEUMONIA.

In cases of pneumonia where the disease is rapidly progressing and for the complications of it, hypodermic injections of ether have a very beneficial effect. The stimulant action is especially noticed in cases of great prostration and where the sputum cannot be ejected from the bronchial tubes. Immediately after the injection is made—the pain which the injection provokes having rapidly subsided—the respiration becomes easy, the pulse is strong and normal, and the tongue gets its natural color and is moist. In time of three minutes the exhalation of ether indicates that it has entered into the lungs, and it takes several hours before the effect is over. The natural crisis is accelerated, and the complications are avoided. The injection is made by the usual Pravay syringe, and one gramme of ether used for one injection. The effects are much quicker than with morphine, and the patient never acquires a use of it like in morphine. It should be applied only in difficult cases causing sometimes paralysis of certain groups of muscles.—*Revista Medico—Quiruraquia.*

